# LPS- induced inflammation exacerbates phospho-tau pathology in rTg4510 mice

**DOI:** 10.1186/1742-2094-7-56

**Published:** 2010-09-16

**Authors:** Daniel C Lee, Justin Rizer, Maj-Linda B Selenica, Patrick Reid, Clara Kraft, Amelia Johnson, Laura Blair, Marcia N Gordon, Chad A Dickey, Dave Morgan

**Affiliations:** 1 Byrd Alzheimer's Institute, Department of Molecular Pharmacology and Physiology, University of South Florida, Tampa, Fl 33612, USA; 2 Byrd Alzheimer's Institute, Department of Molecular Medicine, University of South Florida, Tampa, Fl 33612, USA

## Abstract

Inflammation and microglial activation are associated with Alzheimer's disease (AD) pathology. Somewhat surprisingly, injection of a prototypical inflammatory agent, lipopolysaccharide (LPS) into brains of amyloid precursor protein (APP) transgenic mice clears some of the pre-existing amyloid deposits. It is less well understood how brain inflammation modulates tau pathology in the absence of Aβ. These studies examined the role of LPS-induced inflammation on tau pathology. We used transgenic rTg4510 mice, which express the P301L mutation (4R0N TauP301L) and initiate tau pathology between 3-5 months of age. First, we found an age-dependent increase in several markers of microglial activation as these rTg4510 mice aged and tau tangles accumulated. LPS injections into the frontal cortex and hippocampus induced significant activation of CD45 and arginase 1 in rTg4510 and non-transgenic mice. In addition, activation of YM1 by LPS was exaggerated in transgenic mice relative to non-transgenic animals. Expression of Ser199/202 and phospho-tau Ser396 was increased in rTg4510 mice that received LPS compared to vehicle injections. However, the numbers of silver-positive neurons, implying presence of more pre- and mature tangles, was not significantly affected by LPS administration. These data suggest that inflammatory stimuli can facilitate tau phosphorylation. Coupled with prior results demonstrating clearance of Aβ by similar LPS injections, these results suggest that brain inflammation may have opposing effects on amyloid and tau pathology, possibly explaining the failures (to date) of anti-inflammatory therapies in AD patients.

## Background

Tauopathies consist of intracellular accumulation of the microtubule-associated protein tau in the somatodendritic compartment associated with hyperphosphorylation and aggregation of the protein. Tau dysfunction can lead to neurodegeneration, motor dysfunction, and behavioral deficits in animal models that express mutated forms of the protein [1-4]. One of the most common tauopathies includes Alzheimer's disease (AD). Consequently, numerous studies targeting different components of the disease have been initiated to reduce tau pathology as well as amyloid-β. These include inhibition of kinases which phosphorylate tau, such as glycogen synthase kinase-3β (GSK3β) [5-8], manipulating heat shock proteins [9-11], immunotherapy which targets the tau peptide and subsequently reduces p-tau levels, [12,13], and modifying p-tau by manipulating the immune response [14]. Some reports indicate that pathological tau induces inflammation [15] and that inflammation modifies tau [16,17].

Inflammation arguably plays a significant role in the progression of AD pathology. The microglial activation state contributes to many of the ongoing debates, and it is believed that microglia can cause both beneficial and detrimental effects, depending on the microenvironment and cytokines involved. Recently, more attention has been paid to the functional status of microglia rather than generalized activation by generic markers. As more studies emerge identifying selective markers that represent different phenotypic activation states of microglia, an association of their role during disease pathology is being revealed. In the classical or M1 state, pro-inflammatory cytokines produce tissue damage and pathogen destruction, whereas the alternative activation state (M2) dampens this response and directs tissue repair and healing responses [18]. Some reports suggest that in chronic neurodegenerative diseases like AD, a hybrid activation state exists including markers of both M1 and M2 phenotypes [19]. Other reports argue that beginning stages of AD pathology in animal models of amyloid-β deposition resemble an M2 state that switches to a more classical response with age [20]. It is possible the phenotypic state of microglia in response to amyloid-β deposition influences certain aspects of tau pathology as well. Thus, it is important to identify the components of inflammation that promote vs. reduce tau pathology in order to design better therapeutic strategies which target the immune response.

In previous studies [21-25], intracranial LPS, which induces both M1 and M2 markers, activates microglia and reduces Aβ pathology in APP transgenic models of amyloid deposition. This requires microglial activation and can be suppressed by dexamethasone administered systemically. Importantly, there is no indication for systemic inflammation in AD patients [26]. Herein, we similarly provoked central microglial activation by LPS to evaluate phospho-tau species and pathology in the rTg4510 mice. This model develops tangle pathology in the higher forebrain cortical layers and hippocampus coupled with cognitive deficits and neuronal loss [1,4,27]. To our knowledge, this is the first report showing that activation of inflammation in the brain exacerbates tau phosphorylation.

## Methods

### Mouse breeding, tissue preparations, and animal treatments

The rTg4510 mice, lines carrying the parental tau mutations and the tetracycline-controlled transactivator protein (tTA) were used [3]. For age-related microglia activation, brains were harvested from 1, 5, or 9 month old rTg4510 mice and their non-transgenic littermates. For lipopolysaccharide (LPS) studies, male and female mice were aged 4.5 months and a volume of 2 μl of LPS (5 μg/μl; Salmonella abortus equii, Sigma-Aldrich, St. Louis MO) in was unilaterally injected into the hippocampus and anterior cortex (frontal cortex area3) of rTg4510 and non-transgenic littermates. Stereotaxic coordinates from bregma were +1.7 mm anteroposterior, -2.2 mm lateral and -2.5 mm vertical for frontal cortex, and, -2.7 mm anteroposterior, -2.7 mm lateral and -3.0 mm vertical for hippocampus. The solution was dispensed at a constant rate of 0.5 μl/min. Seven days post injection; mice were weighed and overdosed with 100 mg/kg of pentobarbital. Mice were then perfused intracardially with 25 ml of 0.9% saline. The brain was removed, and immersion fixed in 4% paraformaldehyde in 100 mM PO_4 _buffer (pH 7.4) for 24 hours. The tissue was cryoprotected in a series of 10%, 20% and 30% sucrose solutions. Horizontal sections were cut at 25 μm using a sliding microtome and stored at 4°C in Dulbecco's phosphate buffered saline containing 100 mM sodium azide for immunohistochemistry.

### Immunohistochemistry and silver stain

Immunohistochemistry was performed on free floating sections as described in detail previously [28]. Sections were incubated with primary antibodies rat anti-mouse CD45 (1:3000) (Serotec, Raleigh, NC), rat anti-major histocompatibility complex -II (1:5000) (MHCII; BD Pharmigen), rabbit anti-mouse chitinase 3-like-3 (1:3000) (YM1; StemCell Technologies, Vancouver, Canada), chicken anti-arginase 1 (1:50,000)(generous gift from Dr. S.M. Morris)[29], rabbit anti-human phospho-tau ser199/202 (1:65,000)(Anaspec, Fremont, CA), rabbit anti-human phospho-tau ser396 (1:3000) (Anaspec, Fremont, CA), or rabbit anti- human full length-tau (1:3000) (H-150, sc-5587, Santa Cruz Biotechnology, Santa Cruz, CA), AT8 (1:5000) (Thermo Scientific, Rockford, IL overnight at 4°C, then incubated in the appropriate biotinylated secondary antibody (VectorLabs, Burlingame, CA) for 2 h followed by a 1 hr incubation in ABC (Vector Labs, Birlingame, CA). Color development was performed using 0.05% 3, 3'-diaminobenzidine (DAB; Sigma, St. Louis, MO) enhanced with 0.5% nickelous ammonium sulfate (J. T. Baker Chemical Company, Phillipsburg, NJ). For silver staining, a series of sections stained using Gallyas silver stain method [2]. Briefly, sections were fixed in 4% paraformaldehyde in 100 mM PO_4 _buffer (pH 7.4) for 24 hours, horizontally sectioned at 25 μm thickness, and stored at 4°C in Dulbecco's phosphate buffered saline containing 100 mM sodium azide. Free floating sections were mounted on slides and processed together using Gallyas silver stain method with the omission of a counter stain for quantitative analysis. It should be noted for time courses and LPS studies that all tissue sections for each immunohistochemical stain and the Gallyas silver stain that was analyzed together were processed together at the same time under the same conditions.

### Immunofluorescence

Immunohistochemistry was performed on free floating sections as previously describe above with slight modifications to primary antibody concentrations. Sections were incubated with primary antibodies rat anti-mouse CD45 (1:1000), rabbit anti-human phospho-tau ser199/202 (1:10,000), rabbit anti-mouse chitinase 3-like-3 (YM1) (1:1000), rabbit anti-human phospho-tau ser396 (1:1000), rabbit anti- human full length-tau (1:2000) (H-150), biotinylated AT8 (1:5000) (Thermo Scientific, Rockford, IL) overnight at 4°C, washed and incubated with the appropriate secondary Alexa Fluor antibodies for 2 h (Invitrogen); goat anti-rabbit Alexa 488, goat anti-rat Alexa 488, Streptavidin Alexa 594, donkey anti-chicken Alexa 488, goat anti-rabbit Alexa 594. Sections were mounted on slides with Vectashield, (Vector Labs, Burlingame, CA).

### Image analysis quantification and statistics

Immunohistochemical staining was quantified with Image Pro Plus (Media Cybernetics, Silver Spring, MD) image software. Positively labeled microglia or tau positive neurons were segmented using RGB intensity. Each brain section was imaged at 100× magnification in the anterior cortex centered on the injection site (frontal cortex, area 3), the CA1 or CA3 region of the hippocampus, and entorhinal cortex (caudomedial). Data were obtained as a percent area of the image field that was positively stained by immunochemical or histochemical reaction product. Some sections were digitized on the Zeiss Mirax slide scanner. All values (8 sections) obtained from a single mouse were then averaged to represent a single value for each brain region. Statistical analysis was performed using 2-way ANOVA (Age and Treatment), followed by Fisher's LSD post hoc means comparison test with p values of <0.05 considered significant using Stat View software version 5.0 (SAS Institute Inc, Cary NC). Graphs were generated using GraphPad Prism 4.0 (La Jolla, CA).

## Results

### Age-related CD45 activation in rTg4510 mice

**Figure 1:**
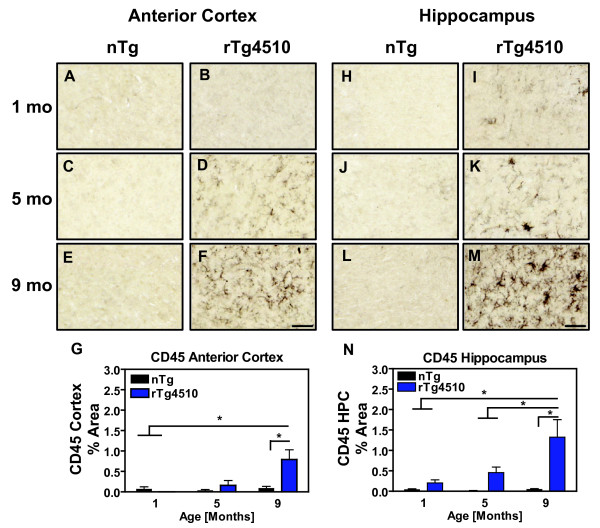
**Age-related, Tau Induced CD45 activation**. Immunohistochemistry was performed for CD45 in rTg4510 and nontransgenic (nTg) littermates aged 1 (A, B, H, I), 5 (C, D, J, K), and 9 (E, F, L, M) months in the anterior cortex (A-F) and hippocampus (H-M). Panels G and N present mean ± S.E.M of % Area for immunostaining of CD45+ microglia in the anterior cortex and hippocampus, respectively. Statistical analysis was performed using 2-way ANOVA followed by Fisher's PLSD multiple comparison test. Area stained for CD45 significantly increased in rTg4510 mice at 9 months of age in the anterior cortex and hippocampus compared with nontransgenic littermates or with younger mice. Lines above relevant bars display significant differences between groups (*p < 0.05), n = 4-5. Sections were digitized and representative images were taken at 40× magnification. Scale bar represents 50 μm.

Previous work has characterized age-related accumulation of various phospho-tau species in forebrain areas and hippocampus of rTg4510 mice [30]. A cross-sectional analysis showed accumulation of insoluble tau species as early as 5.5 months of age. Herein, we evaluated CD45, MHCII and an alternative activation marker, YM1, as markers of microglial activation at 1, 5, and 9 months of age in rTg4510 mice and their non-transgenic littermates. Representative images of anterior cerebral cortex and hippocampus after immunostaining for CD45 are presented in Fig. 1. Neither nontransgenic nor rTg4510 display appreciable staining at one month of age in either cortex (Fig. 1A, B) or hippocampus (Fig. 1H, I). More staining is apparent in sections from older mice (Fig. 1C-F, J-K). Significant activation of CD45 was observed at 9 months in the anterior cortex (Fig.1G) and hippocampus (Fig.1N) compared with age-matched, nontransgenic littermates. Furthermore, CD45 expression of 9 month old rTg4510 mice was significantly greater than observed in either 1 or 5 month old mice in the hippocampus (Fig. 1N) and greater than 1 month old mice in cortex (Fig.1G).

We also examined the microglial markers MHC II and YM-1 as a function of age in rTg4510 mice and their nontransgenic littermates. However, although occasional microglia were positive for MHC II, these were only observed in 9 month old rTg4510 mice (data not shown). We failed to detect any positive YM1 microglia at any age. These data show that age-related accumulation of pathological tau induces CD45 activation in the forebrain of rTg4510 mice.

### LPS induced CD45, YM1 and arginase-1 in rTg4510 mice

**Figure 2:**
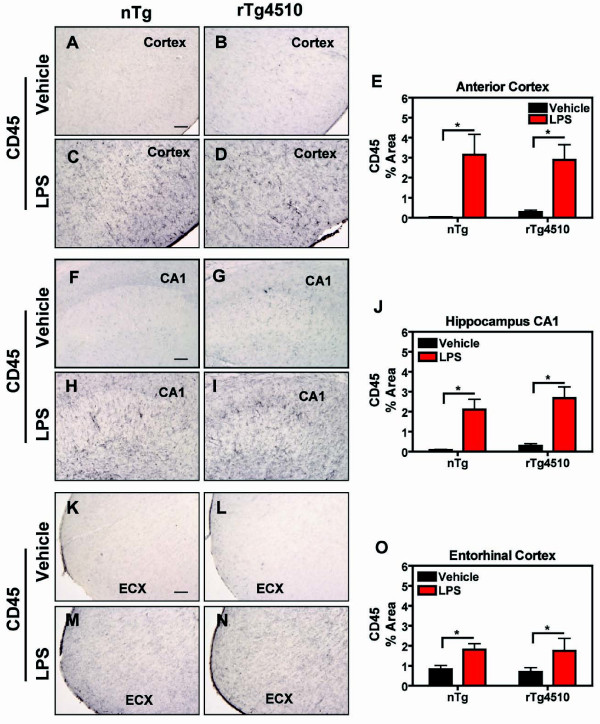
**LPS-Induced CD45 Activation in rTg4510 and nontransgenic mice**. The microglia marker CD45 was examined by immunohistochemical staining in anterior cortex, hippocampus (CA1), and entorhinal cortex (ECX) after LPS administration. rTg4510 mice or nontransgenic (nTg) littermates were injected unilaterally with LPS or vehicle into the anterior cortex and hippocampus, followed by 1 week survival. Representative images from anterior cortex of nontransgenic (A, C) or rTg4510 mice (B, D) from vehicle- (A, B) or LPS-injected (C, D) mice are shown. Images (F-I) were collected from the CA1 stratum radiatum (CA1) of non-transgenic (F, H) or rTg4510 mice (G, I) after vehicle (F, G) or LPS (H, I) injection. Similar images (K-N) were collected from the entorhinal cortex (ECX) of nontransgenic (K, M) or rTg4510 mice (L, N) after vehicle (K, L) or LPS (M, N) injection. Quantitation of % area containing positive immunostaing is presented for each brain region in E, J, and O (mean ± S.E.M, n = 6-8). CD45+ staining increased in both rTg4510 and nontransgenic littermates in anterior cortex, CA1, and ECX of mice treated with LPS compared to vehicle-treated mice. Statistical analysis was performed using 2-way ANOVA followed by Fisher's PLSD multiple comparison test. The asterisk indicates *p < 0.05), n = 6 = 8. Scale bar represents 20 μm.

Previous data show that certain inflammatory events modify the pathology in animal models which deposit amyloid [24]. LPS-induced microglial activation reduces amyloid burdens in the brains of APP Tg2576 mice within one week [23]. We used a similar approach to evaluate tau pathology 1 week following intracranial LPS administration into anterior cortex and hippocampus. LPS injections dramatically induced CD45 activation on the ipsilateral side of the anterior cortex (Fig. 2A-E), hippocampus (Fig. 2F-J), and entorhinal cortex (Fig. 2K-O) compared to vehicle- treated mice in both rTg4510 mice and nontransgenic littermates. Furthermore, significant LPS-induced CD45 activation was also observed on the contralateral side of the cortex and hippocampus compared to vehicle-treated mice (Table 1), although to a lesser extent. The magnitude of LPS-induced CD45 activation was similar in rTg4510 and non-transgenic mice.

**Table 1:** LPS-Induced Microglia Activation on Contralateral Hemisphere

		nTg		rTg4510	
**CD45**	**Region**	**Vehicle**	**LPS**	**Vehicle**	**LPS**

	CX	0.008 ± 0.003	0.397 ± 0.162	0.161 ± 0.110	0.778 ± 0.270 ^*a*^
	CA1	0.027 ± 0.015	0.410 ± 0.100	0.250 ± 0.151	0.489 ± 0.154 ^*a*^
	CA3	0.061 ± 0.020	2.325 ± 0.431	0.112 ± 0.024	1.310 ± 0.527 ^*a*^
	ECX	0.530 ± 0.093	0.920 ± 0.182	0.541 ± 0.118	0.875 ± 0.409
YM1	Region	Vehicle	LPS	Vehicle	LPS

	CX	0.001 ± 0.001	0.024 ± 0.005	0.001 ± 0.001	0.018 ± 0.006 ^*a*^
	CA1	0.003 ± 0.002	0.025 ± 0.007	0.002 ± 0.001	0.100 ± 0.036 ^*a,b*^
	CA3	0.001 ± 0.001	0.049 ± 0.008	0.004 ± 0.002	0.154 ± 0.049 ^*a,b*^
	ECX	0.005 ± 0.002	0.080 ± 0.020	0.001 ± 0.001	0.586 ± 0.189 ^*a,b*^
Arg-1	Region	Vehicle	LPS	Vehicle	LPS

	CX	0.008 ± 0.001	0.012 ± 0.005	0.005 ± 0.001	0.008 ± 0.002
	CA1	0.014 ± 0.002	0.020 ± 0.007	0.008 ± 0.003	0.013 ± 0.003
	CA3	0.017 ± 0.002	0.044 ± 0.024	0.007 ± 0.002	0.047 ± 0.023
	ECX	0.021 ± 0.004	0.039 ± 0.014	0.016 ± 0.005	0.030 ± 0.010

For each marker, 2-way ANOVA follwed by Fisher's LSD Analysis for genotype and treatment were performed. ap < 0.05, Vehicle v.s LPS; bp < 0.05, nTg v.s rTg4510 mice. CD45; microglial activation marker, YM1; microglial alternative activation marker, Arginase-1; alternative activation marker. CX; anterior cortex, CA1 and CA3; hippocampus CA1 and CA3 respectfully, ECX; entorhinal cortex. n = 5-8.

**Figure 3:**
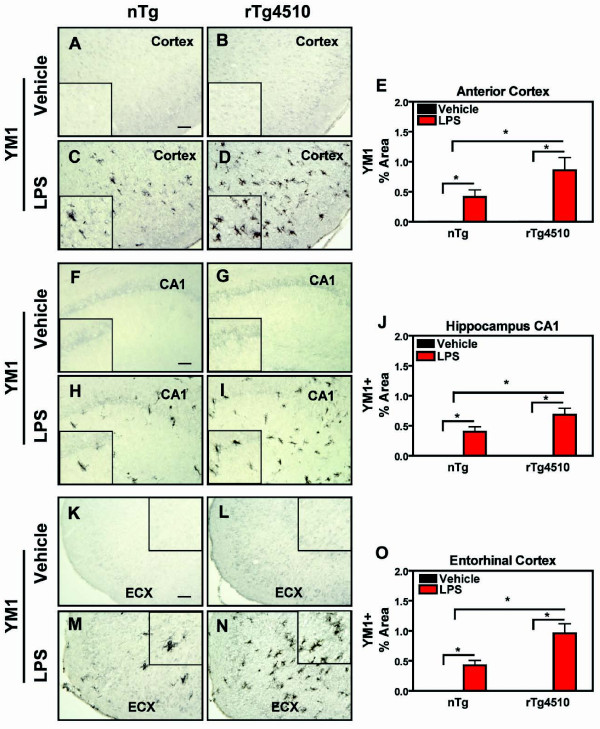
**Exaggerated YM1 Activation in rTg4510 mice**. Immunohistochemical staining for the microglial alternative activation marker YM1 in cortex, hippocampus (CA1), and entorhinal cortex (ECX) was performed. rTg4510 mice or nontransgenic (nTg) littermates were injected with LPS or vehicle into the anterior cortex and hippocampus. Images (A-D) were collected from the anterior cortex of nontransgenic (A, C) or rTg4510 mice (B, D) injected with either vehicle (A, B) or LPS (C, D), from stratum radiatum of CA1 of nontransgenic (F, H) or rTg4510 mice (G, I) after vehicle (F, G) or LPS (H, I) injections, or from entorhinal cortex (ECX) of nontransgenic (K, M) or rTg4510 mice (L, N) which received vehicle (K,L) or LPS (M, N) injections one week previously. Mean ± S.E.M (n = 6-8) of % Area for immunostaining of YM1+ microglia (black) in the anterior cortex, CA1, and ECX are presented in E, J, and O. YM1+ staining increased in CX, CA1, and ECX of mice treated with LPS compared to vehicle-treated mice. Inductions in rTg4510 mice were larger than in nontransgenic littermates. Statistical analysis was performed using 2-way ANOVA followed by Fisher's PLSD multiple comparison test (*p < 0.05), n = 6-8. Scale bar represents 20 μm.

LPS also significantly increased the alternative activation marker YM1 in the ipsilateral hemisphere of the anterior cortex (Fig. 3A-E), hippocampus (Fig. 3F-J), and entorhinal cortex (Fig. 3K-O) compared to vehicle-treated groups in both rTg4510 mice and in nontransgenic littermates. However, unlike CD45 activation, YM1 induction was significantly greater in rTg4510 mice compared to non-transgenic mice. Furthermore, YM1 activation also increased on the contra-lateral hemisphere following LPS (Table 1) and this response was also augmented in the hippocampus and entorhinal cortex of rTg4510 mice compared to nontransgenic littermates.

**Figure 4:**
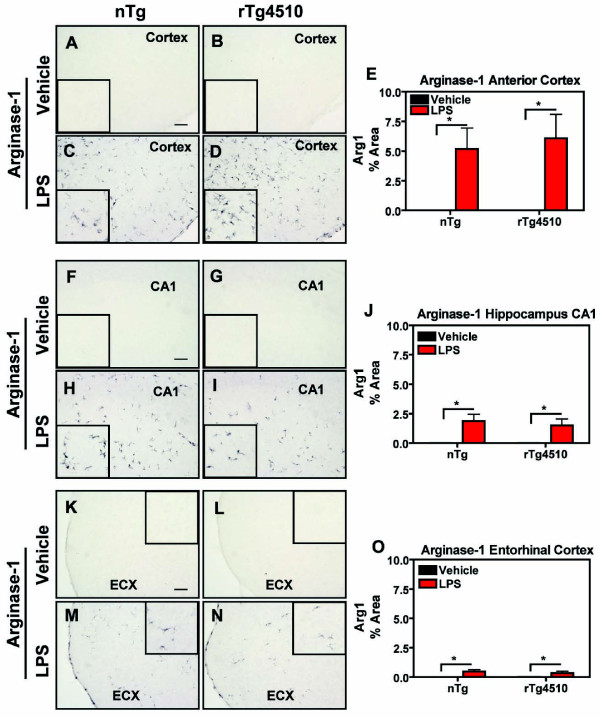
**LPS-Induced Arginase 1 Activation in rTg4510 and nontransgenic mice**. rTg4510 mice or non-transgenic (nTg) littermates were injected with LPS or vehicle, and sections were stained using immunohistochemistry for the microglial marker arginase 1. Digital images from the anterior cortex (A-D) of nontransgenic (A, C) or rTg4510 mice (B, D) injected with vehicle (A, B) or LPS (C, D), from CA1 of nontransgenic (F, H) or rTg4510 mice (G, I) after injections of vehicle (F, G) or LPS (H, I), or entorhinal cortex (ECX) of nontransgenic (K, M) or rTg4510 mice (L, N) after vehicle (K,L) or LPS (M,N) injections are presented. Panels E, J, and O display mean ± S.E.M (n = 6-8) of % Area for immunostaining of arginase 1 positive microglia (black) in the anterior cortex, CA1 CX, and ECX respectively. Arginase 1 staining increased in both rTg4510 and nontransgenic littermates in cortex, CA1, and ECX after treatment with LPS compared to vehicle- treated mice, and the magnitude of the induction was similar in rTg4510 and nontransgenic mice. Statistical analysis was performed using 2-way ANOVA followed by Fisher's PLSD multiple comparison test (*p < 0.05), n = 6-8. Scale bar represents 20 μm.

We also evaluated arginase 1, typically associated with alternative activation. LPS induced robust expression of arginase 1 staining on the ipsilateral side of the anterior cortex (Fig 4A-E), hippocampus (Fig. 4F-J), and entorhinal cortex (Fig. 4K-O) compared to vehicle-treated mice. There was no difference in the size of arginase 1 induction between rTg4510 and nontransgenic mice.

### LPS-Induced inflammation exacerbates phospho-tau pathology

**Figure 5:**
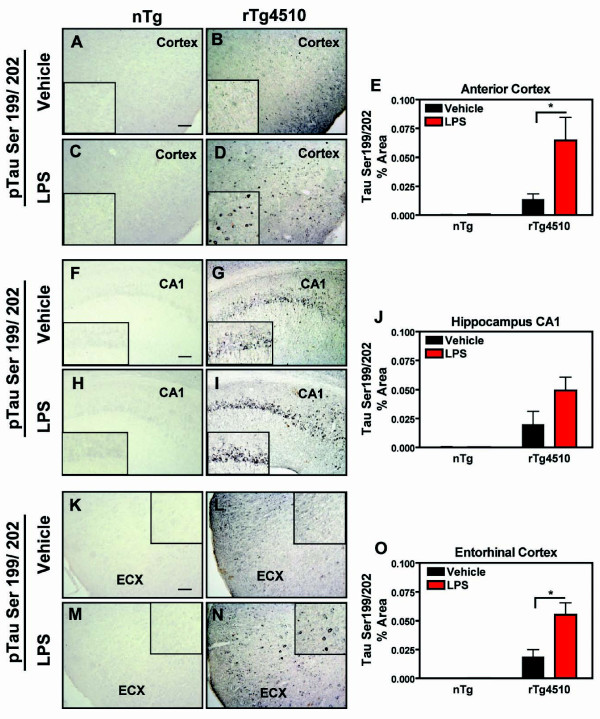
**Increased phosphor-tau staining after LPS**. Immunohistochemical staining of tau phosphorylated at Ser199/202 in anterior cortex (A-D), hippocampus (CA1; F-I), and entorhinal cortex (ECX; K-N) after vehicle (A, B, F, G, K, L) or LPS (C, D, H, I, M, N) administration in rTg4510 (B, D, G, I, L, N) or nontransgenic control mice (A, C, F, H, K, M). Panels E, J and O present mean ± S.E.M of % Area for immunostaining. Phospho-Tau Ser199/202 staining increased in both anterior cortex and ECX of rTg4510 mice treated with LPS compared to vehicle- treated mice. No staining was detected in nontransgenic littermates. Statistical analysis was performed using 2-way ANOVA followed by Fisher's PLSD multiple comparison test. (*p < 0.05), n = 6-8. Scale bar represents 20 μm.

**Figure 6:**
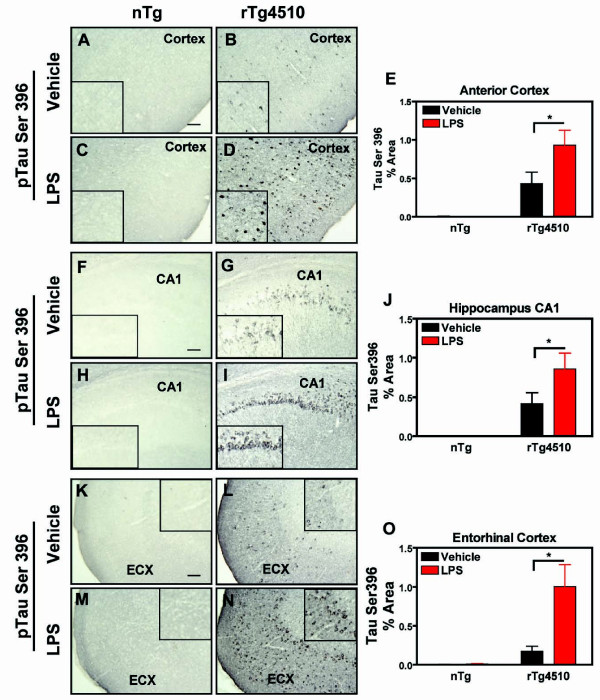
**A second phospho-tau epitope was also increased after LPS**. Immunohistochemical staining of tau phosphorylated at Ser396 in cortex anterior cortex (A-D), hippocampus (CA1; F-I), and entorhinal cortex (ECX; K-N) after injections of either vehicle (A, C, F, H, K, M) or LPS (B, D, G, I, L, N) in nontransgenic (A, C, F, H, K, M) or rTg4510 (B, D, G, I, L, N) mice. The % Area for immunostaining of phospho-tau Ser396 (mean ± S.E.M; n = 6-8) is presented for each brain region in E, J, and O. Phospho-tau Ser396 staining increased in anterior cortex, CA1, and ECX of rTg4510 mice treated with LPS compared to vehicle-treated mice. No staining was detected in nontransgenic littermates. Statistical analysis was performed using 2-way ANOVA followed by Fisher's PLSD multiple comparison test (*p < 0.05), n = 6-8. Scale bar represents 20 μm.

To evaluate the effects of LPS-induced inflammation on tau pathology, we measured pre-tangle pathology by phospho-tau staining, in addition to mature insoluble tangle pathology by Gallyas silver stain. At 5 months, detectable levels of tau phosphorylated at epitope ser199/202 were observed in vehicle- treated rTg4510 mice (Fig. 5B, G, L). LPS significantly increased p-tau ser199/202 in the anterior cortex (Fig. 5A-E) and entorhinal cortex (Fig. 5K-O) in rTg4510 mice compared to the vehicle- treated rTg4510 mice. Although the mean % area for staining of phospho-tau ser199/202 in the hippocampus following LPS administration showed an elevated trend (Fig. 5F-J), it failed to reach statistical significance. Likewise, phospho-tau epitope ser396 was observed in vehicle-treated mice along axonal processes and in perinuclear regions, yet LPS-induced inflammation further increased phospho-tau ser396 immunoreactivity in the cortex (Fig. 6A-E), hippocampus (CA1; Fig. 6F-J), and entorhinal cortex (Fig. 6K-O) compared to rTg4510 mice treated with vehicle. Neither phospho-tau species (tau ser199/202 or ser396) was detectable at the immunohistochemical level in nontransgenic mice which received vehicle or LPS administration.

**Figure 7:**
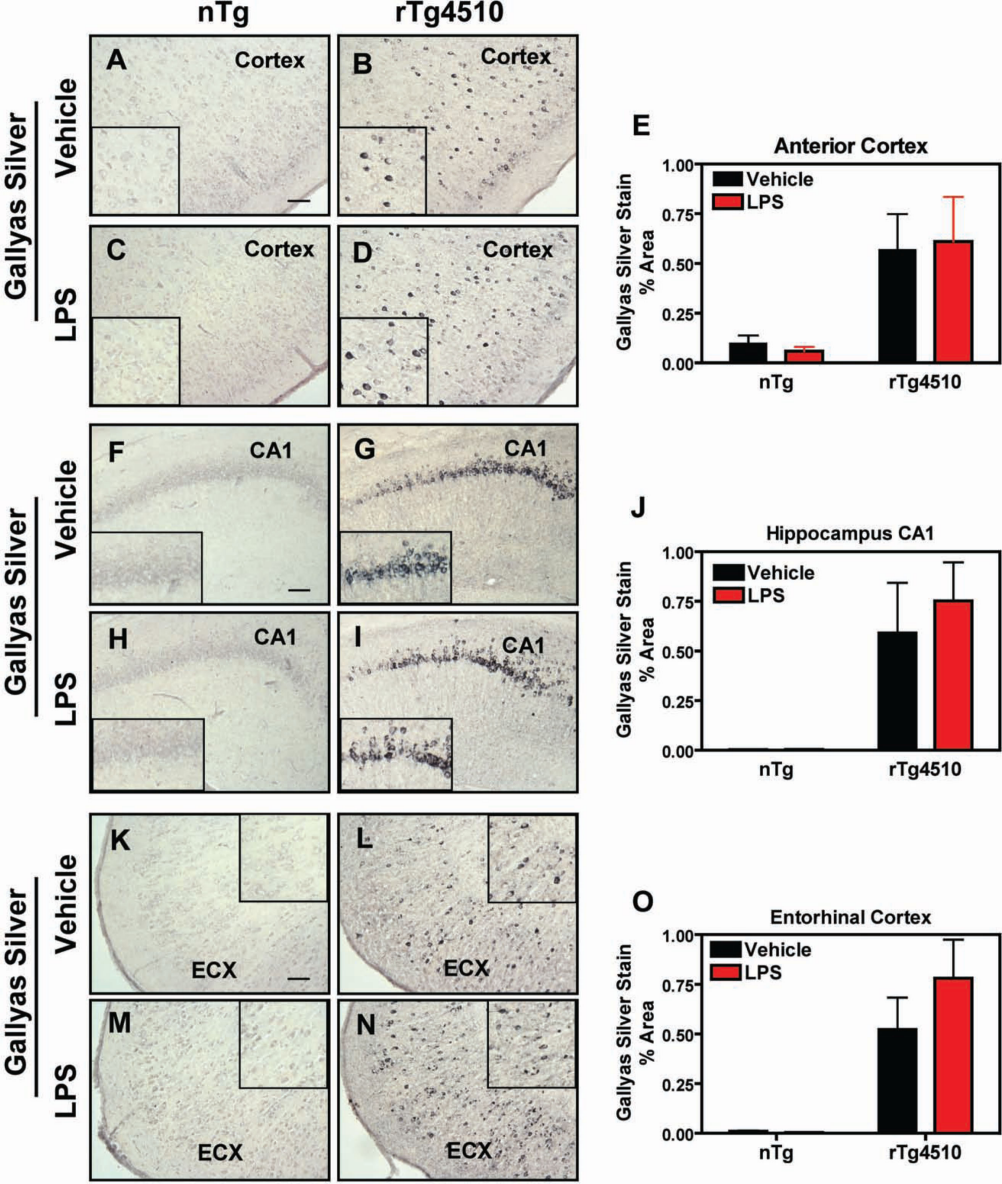
**Argyrophilic tau inclusions did not increase after LPS**. Argyrophilic tau was identified with the Gallyas silver stain in cortex (A-D), hippocampus (CA1; F-I), and entorhinal cortex (ECX; K-N) in nontransgenic (A, C, F, H, K, M) and rTg4510 (B, D, G, I, L, N) mice after injection with LPS (B, D, G, I, L, N) or vehicle (A, C, F, H, K, M). Panels E, J, and O present mean ± S.E.M of % Area for Gallyas silver positive staining. There were no significant changes in silver positive neurons in any brain region of mice treated with LPS compared to vehicle-treated mice. Statistical analysis was performed using 2-way ANOVA followed by Fisher's PLSD multiple comparison test (*p < 0.05), n = 6-8. Scale bar represents 20 μm.

To identify the impact of LPS-induced inflammation on pre-tangle and mature tau pathology, we measured Gallyas silver staining in rTg4510 mice and nontransgenic littermates. Vehicle-treated rTg4510 mice at 5 months of age had small but measurable amounts of Gallyas silver positive neurons. Following LPS administration no significant increases or decreases were observed in the anterior cortex (Fig. 7A-E), hippocampus (Fig. 7F-J), or entorhinal cortex (Fig. 7K-O) of rTg4510 mice. This suggests that acute LPS-induced microglial activation impacts tau phosphorylation but, at least within the first week, does not affect the pre- and mature tangles as determined by Gallyas stain.

**Figure 8:**
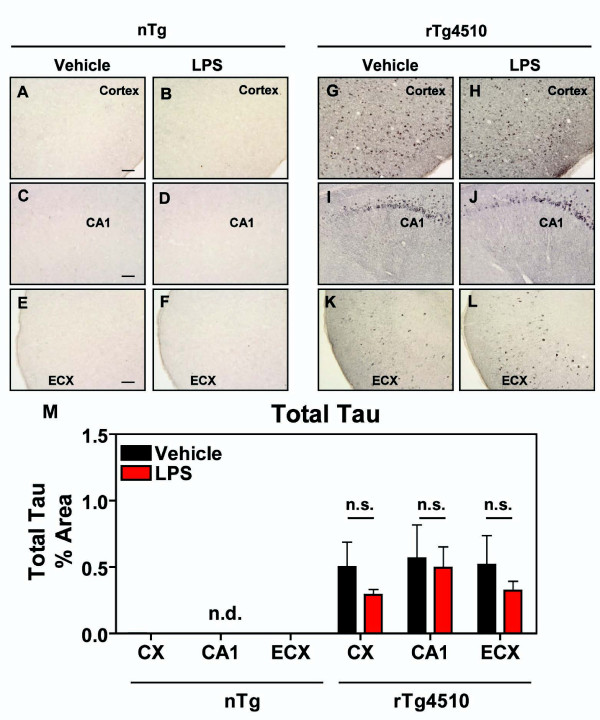
**Total tau was not increased by LPS**. Immunohistochemical staining using an antibody that recognizes multiple isoforms of tau regardless of phosphorylation is presented in anterior cortex (CX; A, B, G, H), hippocampus (CA1; C, D, I, J), and entorhinal cortex (ECX; E, F, K, L) after vehicle (A, C, E, G, I, K) or LPS (B, D, G, H, J, L) administration in nontransgenic (A-F) or rTg4510 (G-L) mice. No immunoreactive staining was observed for total tau in nontransgenic littermates. Mean ± S.E.M (n = 6-8) of % Area for total tau positive staining (M) reveals that there were no significant changes in tau positive staining in the CX, CA1, or ECX of mice treated with LPS compared to vehicle-treated mice. Statistical analysis was performed using 2-way ANOVA followed by Fisher's PLSD multiple comparison test. Scale bar represents 20 μm.

We also evaluated full-length tau in the anterior cortex, hippocampus, and entorhinal cortex by immunohistochemistry (Fig. 8). As observed with the silver stain, tau (H-150) antibody failed to recognize endogenous mouse tau in nontransgenic mice, under these staining conditions at the immunohistochemisrty level (Fig.8A-F). However, detectable levels were observed in rTg4510 mice (Fig. 8G-H) in cortical regions and hippocampus, but were not increased by LPS treatment (Fig. 8M) as was the case for phospho-tau markers potentially due to recognition of multiple isoforms.

### Double labeling of phospho-tau and microglia

**Figure 9:**
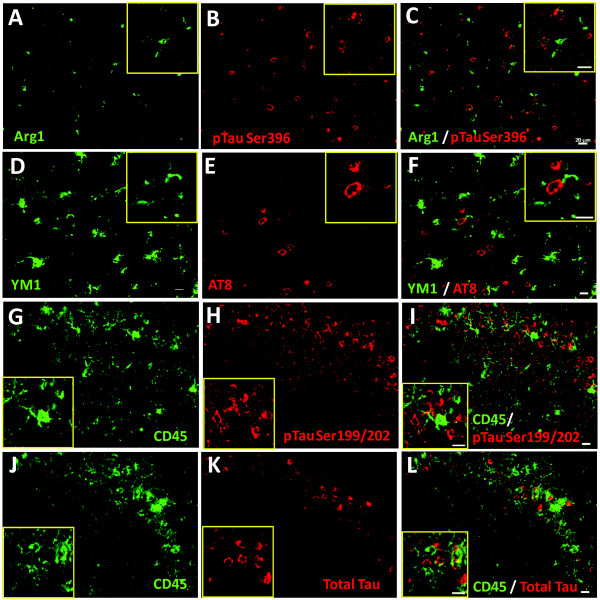
**Immunofluorescent labeling of microglial activation and phospho-tau in rTg4510 mice treated with LPS**. Panels A-C shows immunofluorescent staining of arginase-1 positive microglia (green; A, C) and phospho-tau ser396 (red; B, C) in the anterior cortex. Panels D-F shows YM1 positive microglia (green; D, F) and AT8 (phospho-tauSer202/Thr204) (red; E, F) in the anterior cortex. Panels G-L shows increased CD45 positive microglia (Green; G, I, J, L), phospho-tauSer199/202 (red; H, I), and full-length tau (red; K, L) in the hippocampus CA region. Activated microglia failed to co-label with phospho-tau markers. Images were taken at 20x objective. Scale bar represents 20 μm.

To further identify the relationship between phospho-tau expressing cells and microglia, we performed double labeling studies on rTg4510 mice following LPS injections. Arginase-1 expression was observed in rod and amoeboid-like with some branching cells (Fig. 9A-C) and failed to co-localized with cells stained for phospho-tau Ser396. YM1 positive cells were highly branched and also failed to co-localize with cells stained with the AT8 (phospho-tau Ser202/Thr205) (Fig.9D-F), however several YM1 positive microglia were clustered around AT8 positive neurons. Furthermore, CD45 positive cells displayed various cell morphologies from highly branched to amoeboid/rod and macrophage-like. In general, CD45 activation increased around tau laden areas (phospho-tau Ser199/202 and total tau) such as hippocampus (Fig. 9G-L). Overall, there was rarely cell to cell association and microglia failed to clearly co-localize with phospho-tau positive neurons.

## Discussion

In this study, we show that the phosphorylated tau species previously characterized in the rTg4510 mice [30] are associated with age-related microglial activation as measured by CD45 Further activation of microglia by LPS enhances tau phosphorylation. Prior work [30], demonstrated that young mice possess the ability to clear soluble phospho-tau species, showing reductions in these markers between 1 and 3 months. However, by 5.5 months, insoluble tau aggregates appear in parallel with accumulation of a 64 kD soluble tau species. Thus, microglial activation begins at this age when soluble and insoluble tau species are present. When microglial activation is provoked by LPS challenge at this point, there are clear increases in the phosphorylation of tau. Previous studies showed that LPS-induced microglial activation in APP mice clears amyloid-β pathology in the CNS as early as 3 days following intracranial injection [21-24]. Using this same paradigm, LPS-induced microglial activation in rTg4510 mice exacerbates pre-tangle pathology as visualized by phospho-tau staining. This highlights the need to include mouse models of tau pathology as well as models of amyloid pathology when assessing the impact of potential treatments for translation in to clinical trials in Alzheimer cases.

Another previous study using a 3xTg-AD model (harboring APP_K670N; M671L _, PS1_M146V _, and Tau_p301L _mutations) showed no changes in APP processing after 6 weeks of peripheral administration of LPS. However, phosphorylation of tau at specific sites (AT-180, p231/235; AT8, p202/205; but not PHF-1, p396/404) was increased within the hippocampus, in a cyclin kinase 5-dependent mechanism [16]. Another pro-inflammatory stimulus, interleukin-1β, also resulted in microglial activation and tau phosphorylation in cortical neurons [17]. Herein, we show that acute activation of microglia by LPS increased phospho-tau staining within one week, not only in the hippocampus and anterior cortex, but also in other tau-laden areas that were not injected including entorhinal cortex. Although the level of microglial activation also increased in the entorhinal cortex to a lesser extent than that of the hippocampus and anterior cortex, the increased phospho-tau species observed distal to the injection site is conceivable from previous findings of systemic inflammation and CNS effects on phospho-tau [16] and supports the potential role for diffusible ligands and cytokines and their impact on tau pathology. Although these data suggest that acute inflammatory conditions may accelerate the course of neurodegenerative tauopathies or AD, other models of low level chronic neuroinflammation should be explored in a similar context [31].

With normal aging up to 9 months, CD45 positive microglia increased in parallel with tau pathology, yet the alternative activation marker YM1 was not detected at the protein level by immunohistochemistry. However, upon LPS challenge, YM1 was elevated to a significantly greater level in rTg4510 mice with pre-existing tau than in nontransgenic mice. Thus, not only does microglia activation appear to influence tau pathology but tau pathology appears to impact the phenotypic responses of microglia as well.

It has yet to be determined whether this exaggerated YM1 activation occurred in response to insoluble vs. soluble tau species or even other specific tau forms such as truncated tau. A recent report showed that human misfolded, truncated tau protein promoted the up-regulation of immune molecules in microglia/macrophages and caused the influx of antigen-presenting cells from blood into the CNS of transgenic rats [15]. It is also unknown whether YM1 influences the pathological state of tau, however recent reports show YM1 protein expression localizes around the amyloid plaques of APP/PS1 transgenic mice, and AD brains contain increased YM1 mRNA compared to normal, aged -matched control brains [19,20]. This implies that amyloid pathology can increase YM1 expression. YM1, whose function is poorly understood, exists as a secretory protein transiently expressed in microglia/macrophages during hematopoiesis [32], during parasitic infection or after interleukin-4/interleukin-13 cytokine stimulation [33]. YM1 shows specific binding affinity to glucosamine, galactosamine, and heparin sulfate, which has been hypothesized as a mechanism for shielding or maintaining macrophage integrity during parasitic infection [32,34]. Interestingly, heparin sulfate and sulfated glycosaminoglycans prevent tau from binding to microtubules, promote microtubule disassembly, and stimulate tau phosphorylation by several kinases [35]. Although, LPS induced inflammation and tau pathology seems to influence the expression of YM1, it is unclear if up-regulation of YM1 by cytokines or amyloid-β deposition directly impacts the tau pathology.

Gene delivery of the pro-inflammatory cytokine tumor necrosis factor alpha (TNF-α) into the CNS of 3xTg-AD mice resulted in accumulation of both Aβ42 and phospho-tau species [36]. Conversely, chronic ibuprofen treatment in 3xTg-AD mice reduced oligomeric amyloid-β, hyperphosphorylated tau, and improved memory deficits [37]. However, overexpression of the pro-inflammatory and M1-stimulating cytokine, interferon gamma, using AAV resulted in differential effects on amyloid-β and phospho-tau[14]. The authors observed increased levels of amyloid-β but reduced levels of phospho-tau, contradicting results with INF-γ in 3xTg-AD mice [14]. Given the evidence that the amyloid deposition drives tau pathology in this 3xTg-AD model [12], it is unclear whether direct effects on tau or indirect effects on amyloid are responsible for changes in tau pathology.

Transgene regulated over-expression of the M1-stimulating cytokine, interleukin-1 in APP mice caused reductions in amyloid pathology [38]. Similarly, AAV-mediated over-expression of interleukin-6 in TgCRND8 and Tg2576 mice elicited massive gliosis and reductions in amyloid-β pathology [39]. The microglial phenotype in these mice included increased YM1, but not other M2 activation markers such as arginase 1. In our study, we observed a tau transgene facilitation of YM1 induction following LPS in both hemispheres, but this effect was not evident for arginase 1activation, another putative M2 activation marker. This raises questions regarding how particular M2 activation state markers are regulated by proinflammatory stimuli and why RNA and protein levels of YM1 are increased in AD patients and animal models of amyloid deposition, which are typically considered to be associated with a proinflammatory (M1) cytokine environment [19,20]. These observations are important in considering the ultimate goals for therapeutic tuning of the microglial phenotype in order to reduce amyloid and/or tau pathology. Some dichotomous effects in the different transgenic models and therapeutic treatments make interpretations and potential translation to AD challenging. These results suggest a more complex set of microglia phenotypes than the dipolar M1/M2 characterization, and suggest that the clearance of amyloid pathology and tau pathology may be mediated by distinct activation subtypes.

## Abbreviations

AD: Alzheimer's disease; LPS: lipopolysaccharide, APP: amyloid precursor protein; AAV: adeno-associated virus

## Competing interests

The authors declare that they have no competing interests.

## Authors' contributions

DCL led the study and involved in all aspects from surgeries to analysis of data, and manuscript preparation. JR participated in surgeries, immunohistochemical staining, and data acquisition. MS and PR participated in tissue preparation, immunohistochemical/fluorescent staining and data acquisition, generation of figures. CK, AJ, and LB performed Gallyas Silver staining and maintained animal husbandry, breeding, and genotyping of mice and data collection. CD, MG, DM were involved in overall conception, secured funding for the project, experimental design, manuscript preparation and analysis. All authors have read and approved the final manuscript.
